# AGE DIFFERENCES IN EXPOSURE TO NOVEL SITUATIONS IN DAILY LIFE AND ASSOCIATED EMOTIONAL EXPERIENCE

**DOI:** 10.1093/geroni/igac059.1895

**Published:** 2022-12-20

**Authors:** Li Chu, Yochai Shavit, Nilam Ram, Laura Carstensen

**Affiliations:** Stanford University, Menlo Park, California, United States; Stanford Center on Longevity, Stanford University, Stanford, California, United States; Stanford University, Stanford, California, United States; Stanford University, Stanford, California, United States

## Abstract

Exposures to novelties are cognitively beneficial in later adulthood, but their impact on emotional well-being is still unknown. Novel situations may bring excitement as well as anxiety, and this may be different across ages According to socioemotional selectivity theory, older adults prioritize familiar and positive experiences that likely contribute to better emotional well-being over novel and negative ones, suggesting that older adults may be motivated to avoid novel situations, especially if these experiences are associated with negative emotions. This study examined age differences in novelty experienced in daily life and the associated emotions. We utilized experience sampling data collected five times a day for one week from 375 participants (age range=18-94). Contrary to the hypothesis, older age exhibited a quadratic association with novel daily experiences such that situation novelty was lowest in middle adulthood. Results from multilevel models suggested that people with higher overall exposure to novel situations had higher overall levels of negative emotions, and that when in more novel situations the prototypical individual experienced more negative emotions in general. However, consistent with SST postulates, one of these associations were moderated by age; older adults experienced lower positive emotions during novel situations than younger individuals. In contrast, the association between situation novelty and negative emotions did not differ with age. Together, these findings suggest that older adults do often find themselves in novel situations but may experience them less positively than younger adults. Implications on learning-related programs and interventions designed to expose older adults to novelties will be discussed.

